# Predicting survival times for neuroblastoma patients using RNA-seq expression profiles

**DOI:** 10.1186/s13062-018-0213-x

**Published:** 2018-05-30

**Authors:** Tyler Grimes, Alejandro R. Walker, Susmita Datta, Somnath Datta

**Affiliations:** Department of BiostatisticsUniversity of Florida, 2004 Mowry Rd, Gainesville, 32611 USA

**Keywords:** Accelerated failure time, Sparse PLS, Lasso, Elastic net, Data imputation

## Abstract

**Background:**

Neuroblastoma is the most common tumor of early childhood and is notorious for its high variability in clinical presentation. Accurate prognosis has remained a challenge for many patients. In this study, expression profiles from RNA-sequencing are used to predict survival times directly. Several models are investigated using various annotation levels of expression profiles (genes, transcripts, and introns), and an ensemble predictor is proposed as a heuristic for combining these different profiles.

**Results:**

The use of RNA-seq data is shown to improve accuracy in comparison to using clinical data alone for predicting overall survival times. Furthermore, clinically high-risk patients can be subclassified based on their predicted overall survival times. In this effort, the best performing model was the elastic net using both transcripts and introns together. This model separated patients into two groups with 2-year overall survival rates of 0.40±0.11 (*n*=22) versus 0.80±0.05 (*n*=68). The ensemble approach gave similar results, with groups 0.42±0.10 (*n*=25) versus 0.82±0.05 (*n*=65). This suggests that the ensemble is able to effectively combine the individual RNA-seq datasets.

**Conclusions:**

Using predicted survival times based on RNA-seq data can provide improved prognosis by subclassifying clinically high-risk neuroblastoma patients.

**Reviewers:**

This article was reviewed by Subharup Guha and Isabel Nepomuceno.

## Background

Neuroblastoma is the most frequently diagnosed cancer in the first year of life and the most common extracranial solid tumor in children. It accounts for 5% of all pediatric cancer diagnoses and 10% of all pediatric oncology deaths [1]. These numbers have improved over the past decade, but accurate prognosis for the disease has remained a challenge [1]. The difficulty is due to the highly heterogeneous nature of neuroblastoma; cases can range from tumors that spontaneously regress on their own, to aggressive tumors that spread unabated by treatment.

In 1984, the MYCN oncogene was identified as a biomarker for clinically aggressive tumors [2]. It has since been one of the most important markers for stratifying patients. Genome-wide association studies have found many other SNPs associated with an increased risk of neuroblastoma. However, while aberrations of these genes indicate an increased susceptibility to the disease, these markers are less useful for stratifying patients into risk groups after diagnosis.

The Children’s Oncology Group stratifies patients into three risk groups using the International Neuroblastoma Staging System (INSS) and various prognostic markers including age at diagnosis, tumor histology, MYCN amplification, and DNA ploidy. According to the American Cancer Society, the 5-year survival rate for these low-risk, intermediate-risk, and high-risk groups are >95*%*, 90% - 95%, and < 50%, respectively. The high-risk group typically consists of patients older than 18 months with INSS stage 4 or patients of any age with MYCN amplification.

Predicting survival outcomes using gene expression data has been explored with promising results [3, 4]. These studies use gene expression profiles with classification methods to stratify patients into risk groups. However, patients that are clinically labeled as high-risk pose a particular challenge, and classifiers tend to struggle separating those patients into subgroups. In this paper, we take the approach of modeling survival time directly using RNA-seq data. This leads to two objectives: the first is to evaluate the accuracy of the model in predicting exact survival times. The second is to determine whether the predicted times can be used to subclassify high-risk patients into distinct groups.

## Methods

### Accelerated failure time (AFT) model

The accelerated failure time (AFT) model relates the log survival times to a linear combination of the predictors.

1 $$ \log(y) = X\beta + \epsilon,  $$

where $y \in R^{+^{n}}$ denotes the vector of *n* observed survival times, *X* the *n*×*p* matrix with columns containing the predictor variables for each observation, *β*∈*R*^*p*^ the vector of regression coefficients, and *ε*∈*R*^*n*^ a vector of independent random errors with an unspecified distribution that is assumed to be independent of *X*. The predictors *X* are centered and scaled so that each column *X*_*i*_, for *i*=1,…,*p*, has zero mean and unit variance There are two challenges to fitting this model: the high dimensionality of *X* and the right censoring of *y*. Since *p*>*n*, ordinary least squares (OLS) should not be used as it will simply overfit on the data. Instead, four approaches for dimension reduction are considered, which include both latent factor and regularization techniques. To handle right censoring, a nonparametric, iterative imputation procedure is proposed, which allows the model to be fit as though complete data were available.

Each of the dimension reduction techniques require the selection of one or more tuning parameters. These parameters are determined by 10-fold cross validation, which is implemented in R using two packages discussed in the following sections.

#### PLS

With partial least squares (PLS), a collection of *v*<*n* orthogonal latent factors are computed as linear combinations of the original covariates. The construction of the latent factors takes into account both *X* and *y*; this is in contrast to principal component analysis (PCA), which only considers *X*. A review of PLS and its application to genomic data can be found in [5]. Once the *v* latent factors are computed, the AFT model is fit using OLS with these new variables.

PLS is implemented using the “spls” R package [6]. The number of latent factors *v* is a tuning parameter, which is determined from 10-fold cross validation. The optimal value of *v* is searched over *v*=1,…,10.

#### SPLS

Like PLS, the sparse partial least squares (SPLS) also constructs latent factors, but it incorporates *L*_1_ regularization in the process [7]. This induces sparsity in each linear combination of the original covariates that make up the latent factors. There are two tuning parameters, the number of latent factors *v*<*n* and the shrinkage parameter *η*∈(0,1) for the regularization. Both of these are determined from 10-fold cross validation using the “spls” R package [6]. The optimal values of *v* and *η* are searched over the grid of points with *v* = 1,.., 10 and *η*=0.1,…,0.9.

Note, to implement PLS the shrinkage parameter, *η*, is set to zero.

#### Lasso

The least absolute shrinkage and selection operator (lasso) fits the model using least squares subject to an *L*_1_ constraint on the parameters $\sum _{j = 1}^{p} |\hat {\beta }_{j}| \leq \lambda $, where *λ*>0 is a tuning parameter that affects the amount of shrinkage [8]. This constraint induces sparsity in the estimated coefficients, setting many coefficients to zero and shrinking others.

The model is fit using the “glmnet” R package [9], which performs 10-fold cross validation to select *λ*.

#### Elastic net

The elastic net (elnet) uses a similar approach as the lasso. It combines both *L*_1_ and *L*_2_ penalties; the estimator minimizes the convex function

2 $$ \frac{1}{2} ||Y - X\beta||_{2}^{2} + \lambda\left[ \frac{1}{2}(1 - \alpha)||\beta||_{2}^{2} + \alpha||\beta||_{1}\right],  $$

where *λ*>0 and *α*∈ [ 0,1] are two tuning parameters [10]. When *α*=1, this reduces to the lasso. By including some component of the *L*_2_ penalty, groups of strongly correlated variables tend to be included or excluded in the model together. The “glmnet” R package [9] is used to fit the model and determine both tuning parameters.

### Imputation for right censoring

Let {(*y*_*i*_,*δ*_*i*_,*X*_*i*_)|*i*=1,…,*n*} denote the set of observed survival times, indicators for death from disease, and the *p*-dimensional vector of covariates for the *n* patients in the dataset. Let *T*_*i*_ denote the true survival times for patient *i*=1,…,*n*. If the *i*th patient’s survival time is censored (i.e. *δ*_*i*_=0) then we only observe *y*_*i*_<*T*_*i*_. That is, *T*_*i*_ is unobserved.

To deal with this right censoring, the dataset imputation procedure from [11] is used. This procedure is briefly summarized here. To begin, an initial estimate $\hat {\beta }^{(0)}$ is obtained by fitting the AFT model using only the uncensored data. Then, in each of *k*=1,…,*n*_*K*_ iterations, do the following.

1. Calculate the Kaplan-Meier estimate $\hat {S}^{(k)}(e)$ of the distribution of model error using {(*e*_*i*_,*δ*_*i*_)|*i*=1,…,*n*} where $e_{i} = \log (y_{i}) - X_{i}^{T}\hat {\beta }^{(k - 1)}$.
2. Impute *n*_*D*_ new datasets by replacing each censored log(*y*_*i*_) with $X_{i}^{T} \hat {\beta }^{(k - 1)} + e_{i}^{*}$, where $e_{i}^{*}$ is a sampled model residual from the conditional distribution $\hat {S}^{(k)}(e | e > e_{i})$. This condition ensures that the imputed observation will be larger than the observed right-censored time.
3. Use the new datasets to compute *n*_*D*_ new estimates $\tilde {\beta }_{j}^{(k)}$ for *j*=1,…,*n*_*D*_.
4. Average the *n*_*D*_ estimates to obtain a final estimate $\hat {\beta }^{(k)} = \frac {1}{n_{D}} \sum _{j=1}^{n_{D}} \tilde {\beta }_{j}^{(k)}$.

The process is repeated for *n*_*K*_ iterations, and the final estimate $\hat {\beta }^{(n_{K})}$ is returned.

To balance between computation time and simulation variability, we chose to run *n*_*K*_=5 iterations, imputing *n*_*D*_=5 datasets in each.

### Ensemble method

The ensemble method incorporates bagging with rank aggregation over each performance measure. The 12 models using genes, transcripts, and introns each with PLS, SPLS, lasso, and elnet are considered, along with the clinical data only model. These 13 models are combined using the ensemble method presented in [12], which is briefly summarized here.

For *i*=1,…,*B* iterations, do the following

1. From the original training dataset, resample *n* observations with replacement. This set is referred to as the bag and will be used to train the ensemble. The out of bag (OOB) samples consist of those not chosen for the bag and are used to test the ensemble.
2. Each of the *M*=13 models are fit on the bag samples.
3. Compute *K* performance measures for each model using the OOB samples.
4. The models are ordered $R^{i}_{(j)}$, for *j*=1,…,*M*, by rank aggregation of the *K* measures. The best model $R^{i}_{(1)}$ is collected.

This process results in a collection of *B* models. The ensemble method uses the average of the predicted survival times from each of these *B* models.

In this study, we consider *K*=3 different measures: the RMSE and two logrank test statistics described below. A total of *B*=20 iterations are conducted, which keeps the computational burden at a minimum while maintaining desirable results. In addition, to avoid repeating the imputation procedure for each model at each iteration, the censored data is imputed once at the start of the ensemble training; the censored survival times are replaced with the predicted times from the single best model (TI-4).

### Classification: LPS vs. non-LPS

The second goal is to subclassify clinically high-risk patients. A new dichotomous variable is created to classify patients: If the predicted survival time is less than *t*>0 years, we say the patient has low predicted survival (LPS). Otherwise, the patient is non-LPS. For patient *i*=1,...,*n* with predicted survival time $\hat {y}_{i}$, let

3 $$ \text{LPS}_{i, t} = \left\{ \begin{array}{cc} 1 & \,\,\text{if} \ \hat{y}_{i} \leq t \\ 0 & \,\,\text{otherwise} \end{array} \right..  $$

Two cutoffs were considered with *t*=2 and *t*=5 years. For clinically high-risk patients, the *t*=2 cutoff is useful for identifying those with a significantly lower survival rate. In the general population of neuroblastoma patients, the *t*=5 cutoff is useful as an alternative way to identify high-risk patients, but it cannot tease out the more extreme cases.

### Performance measures

Performance is evaluated on the testing dataset by four different measures.

The first involves the prediction error of survival times. This is measured by the root mean squared error, adjusted to account for the censoring by reweighting each error by the inverse probability of censoring [13]. This is given by,

4 $$ \text{RMSE} = \left(\frac{1}{n} \sum_{i = 1}^{n} \frac{\delta_{i} \left(y_{i} - \hat{y}_{i}\right)^{2}}{\hat{S}^{C}\left(T_{i}^{C}-\right)}\right)^{1/2},  $$

where *n* is the sample size of the testing dataset, *δ*_*i*_ is 1 if the *i*th patient is uncensored and 0 otherwise, *y*_*i*_ is the observed survival time for patient *i*, $\hat {y}_{i}$ is the predicted survival time, and $\hat {S}^{C}$ is the survival function of censoring. Note that $\hat {S}^{C}$ can be estimated by the Kaplan-Meier estimator with *δ* replaced by 1−*δ*.

A reviewer suggested Harrell’s c-index as an alternative measure to RMSE. The c-index measures the concordance of predicted survival times with true survival times. It is computed as

5 $$ \hat{C}_{H} = \frac{\sum_{i \neq j} \delta_{i} I\left(\hat{y}_{i} < \hat{y}_{j}\right) I\left(y_{i} < y_{j}\right)}{\sum_{i \neq j} \delta_{i} I(y_{i} < y_{j})}.  $$

In contrast to RMSE, the c-index only considers the relative ordering of the predicted times. The c-index ranges from 0 to 1, with values close to 1 indicating strong performance.

The final two measures are based on the LPS classification of patients using cutoffs *t*=2 and *t*=5. A model is considered to peform well if it is able to separate patients into two groups having distinctly different survival curves. To measure this property, the logrank test [14] is used, which compares the estimated survival curves for each group (LPS versus non-LPS). The test statistic is given by

6 $$ \frac{\left(O_{g} - E_{g}\right)^{2}}{\text{Var}\left(O_{g} - E_{g}\right)},  $$

where $O_{g} - E_{g} = \sum _{f \in F} \left (d_{g, f} - d_{f} (n_{g, f} / n_{f})\right)$ is the sum of observed minus expected deaths in group *g*=1,2, where *F* is the set of all observed survival times, *d*_*g*,*f*_ is the number of deaths in group *g* at time *f*, *n*_*g*,*f*_ is the number of patients at risk in group *g* at time *f*, and *n*_*f*_ is the total number at risk at time *f*. The survdiff function in the “survival” R package [15] is used to compute this statistic. Under the null hypothesis of no difference between survival curves, the logrank test statistic has an asymptotically *χ*^2^ distribution with 1 degree of freedom.

**Fig. 1:**
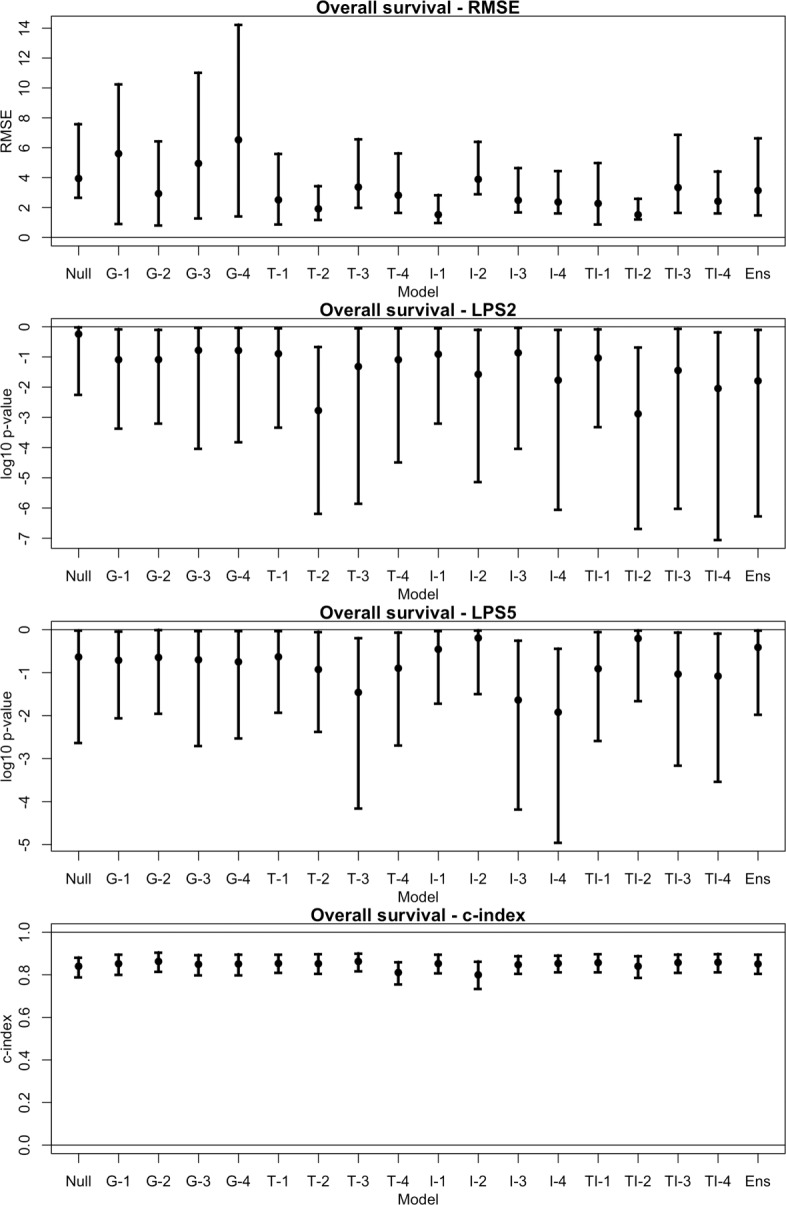
Performance measures for overall survival. Each of the 18 models are assessed using the testing dataset. Four measures of performance are considered: the adjusted root mean squared prediction error (RMSE); the logrank test statistic from using the predicted survival time as a classifier on high-risk patients, thresholded at 2 years (LPS2) and 5 years (LPS5); and Harrell’s c-index. 95% confidence intervals are obtained by bootstraping on the testing dataset. This is done by resampling observations with replacement and recomputing each measure. The process is repeated for *B*=1000 times, and the middle 95% of measures are used for the confidence interval

**Fig. 2:**
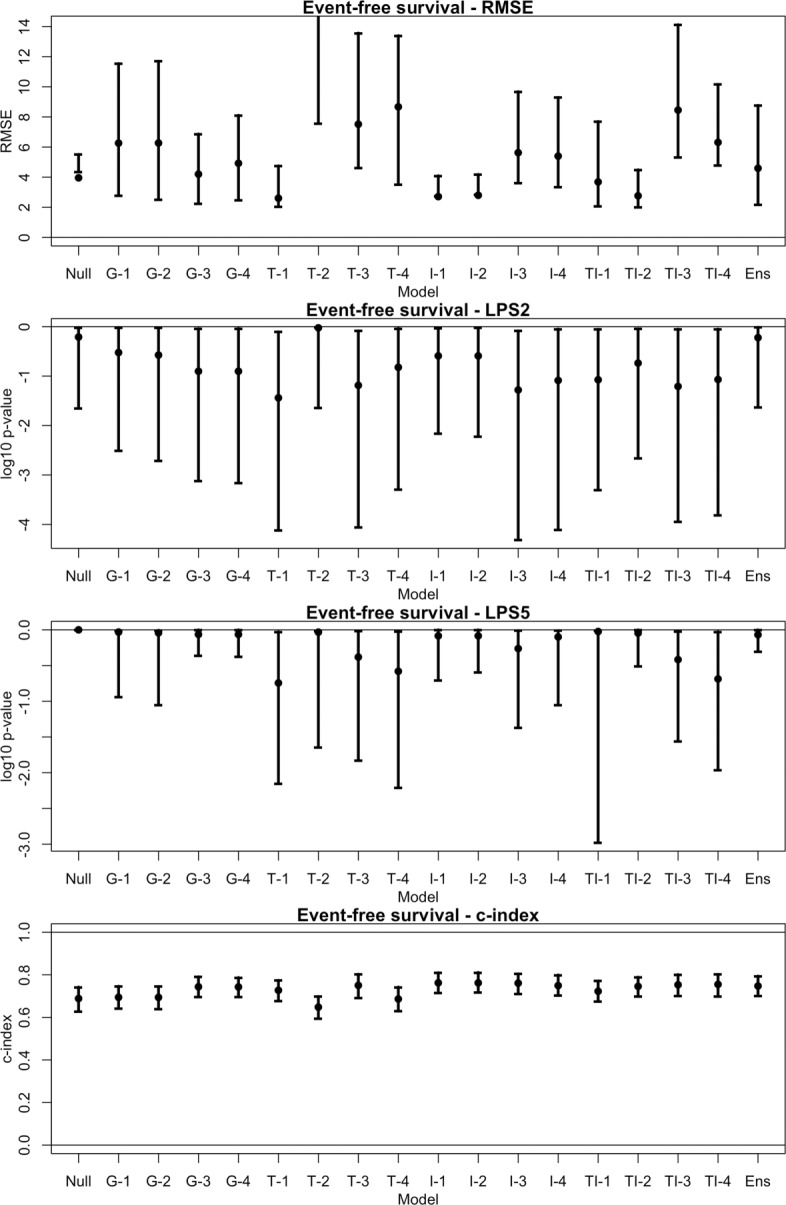
Performance measures for event-free survival. Each of the 18 models are assessed using the testing dataset. Four measures of performance are considered: the adjusted root mean squared prediction error (RMSE); the logrank test statistic from using the predicted survival time as a classifier on high-risk patients, thresholded at 2 years (LPS2) and 5 years (LPS5); and Harrell’s c-index. 95% confidence intervals are obtained by bootstraping on the testing dataset. This is done by resampling observations with replacement and recomputing each measure. The process is repeated for *B*=1000 times, and the middle 95% of measures are used for the confidence interval. Note, the upper limit of RMSE for T-2 is not visible in the plot

The performance measures for each model are shown in Figs. 1 and 2. For RMSE and the logrank tests, smaller values correspond to better performance. For c-index, values close to 1 are better. The error bars are 95% confidence intervals obtained by bootstraping on the testing dataset; observations are resampled with replacement and each measure is recomputed. The process is repeated *B*=1000 times. The 2.5th and 97.5th percentiles are used for the lower and upper confidence limits, respectively.

### Datasets

The datasets can be accessed from the GEO database with accession number GSE49711 [16, 17]. The data are comprised of tumor samples from 498 neuroblastoma patients from seven countries: Belgium (*n*=1), Germany (*n*=420), Israel (*n*=11), Italy (*n*=5), Spain (*n*=14), United Kingdom (*n*=5), and United States (*n*=42). Several clinical variables are available for each patient, along with the RNA-sequencing information from their tumor sample. In [16], the data were randomly separated into a training set and testing set; this partition was recorded with the clinical data and is used here.

#### Clinical data

The clinical data consist of 11 variables. In this study, three of these variables are used as clinical covariates: sex, age, and MYCN status.

There are two outcomes of interest: overall survival and event-free survival. Overall survival is calculated as the time from diagnosis to the time of death from disease or the last follow-up date, if the patient survived. Event-free survival is calculated as the time from diagnosis to the time of tumor progression, relapse, or death from disease, or to the last follow-up date if no event occurred.

#### RNA-seq data

The RNA-seq data provide annotations at three feature levels, giving datasets comprised of 60,776 genes, 263,544 transcripts, and 340,414 introns, respectively. A hierarchical version of the transcript annotation was also available but was not used.

Normalization of the RNA-seq data was performed by [16]. The gene counts were normalized as the log2 of the number of bases aligned in the gene, divided by the number of terabases aligned in known genes and by the length of the gene, with several corrections. The same normalization is used for the transcript counts. The expressions for the introns are computed as

$${}\log_{2}\frac{(1 + \text{number of supporting reads})*10^{6}}{\text{number of reads supporting an intron in this data}}. $$

The RNA-seq data are filtered prior to model fitting. Genes and transcripts without an NCBI ID are removed. Any variables with over 80% zero counts in the training dataset are also omitted. A database of 3681 genes related to neuroblastoma was obtained from the GeneCards Suite [18]. This dataset is used to subset the remaining genes and transcripts, resulting in 3389 genes and 47276 transcripts. For the introns, their predictive ability for survival time is ranked by fitting each intron in a Cox proportional hazards model [19, 20]. This is repeated for both OS and EFS times of patients in the training set. The Cox model is fit using the “survival” R package [15]. The top 2000 introns with the smallest *p*-values (testing that the coefficient is zero) are used. This ranking is also performed on the remaining genes and transcripts; the top 2,000 of each are retained.

## Results

Eighteen models are considered in total. Each model is used to estimate overall survival (OS) and event-free survival (EFS). For a baseline of comparison, a “null” model is fit using clinical covariates alone. Models are then constructed by first selecting a set of predictors: genes, transcripts, introns, or both transcripts and introns (labeled G, T, I, and TI, respectively); and then choosing one of the four dimension reduction techniques: PLS, SPLS, lasso, or elastic net (labeled 1-4, respectively). This gives 16 possible combinations. Finally we consider an ensemble model, which pools together the null model and individual models containing genes, transcripts, or introns.

### Predicting survival times directly

The models using RNA-seq data tend to perform better than the null model in predicting survival times. A 95% confidence interval (CI) for the adjusted root mean squared error (RMSE) of each model is estimated via bootstrapping on the testing set; these are shown in Figs. 1 and 2.

For OS, the estimated 95% CI for RMSE of the null model is (2.66, 7.61). Every other model besides G-1, G-3, and G-4 (genes using PLS, lasso, and elnet, respectively) have smaller RMSE estimates than the null model. However, only the TI-2 model (transcripts and introns using SPLS) has a confidence interval bounded below the null model’s, with an estimated 95% CI of (1.23, 2.60) (Fig. 6). For EFS, the improvements of the RNA-seq models over the null model appear to be less substantial. The estimated 95% CI for RMSE of the null model is (4.37, 5.52). Only five of the 16 RNA-seq models have lower RMSE estimates than the null model. The TI-2 model still performed well in comparison with a 95% CI for RMSE of (2.02, 4.49), which overlaps slightly with the null model’s. The I-1 and I-2 models (introns using PLS and SPLS) have confidence intervals bounded below the null model’s (Fig. 7).

Overall, the performance of predicting exact survival times is not completely satisfactory. For a patient with high predicted survival, say 20 years or more, an RMSE of 1-2 years is acceptable; we can reliably conclude that this is a low-risk patient who won’t require intensive treatment. However, a clinically high-risk patient may have a predicted survival time of 5 years or less, in which case an RMSE of 1-2 years is troublesome; it is unclear whether or not an agressive course of treatment should be used.

A reviewer suggested the use of Harrell’s c-index as an alternative measure to RMSE. This measure considers the relative ordering of predicted survival times with the observed times [21]. We find that models provide predicted times that are strongly concordant with observed times (Figs. 1 and 2), which indicates an accurate relative ordering of patients. These results suggests that the models may be useful as a classifier.

### Classification of high-risk patients

**Fig. 3:**
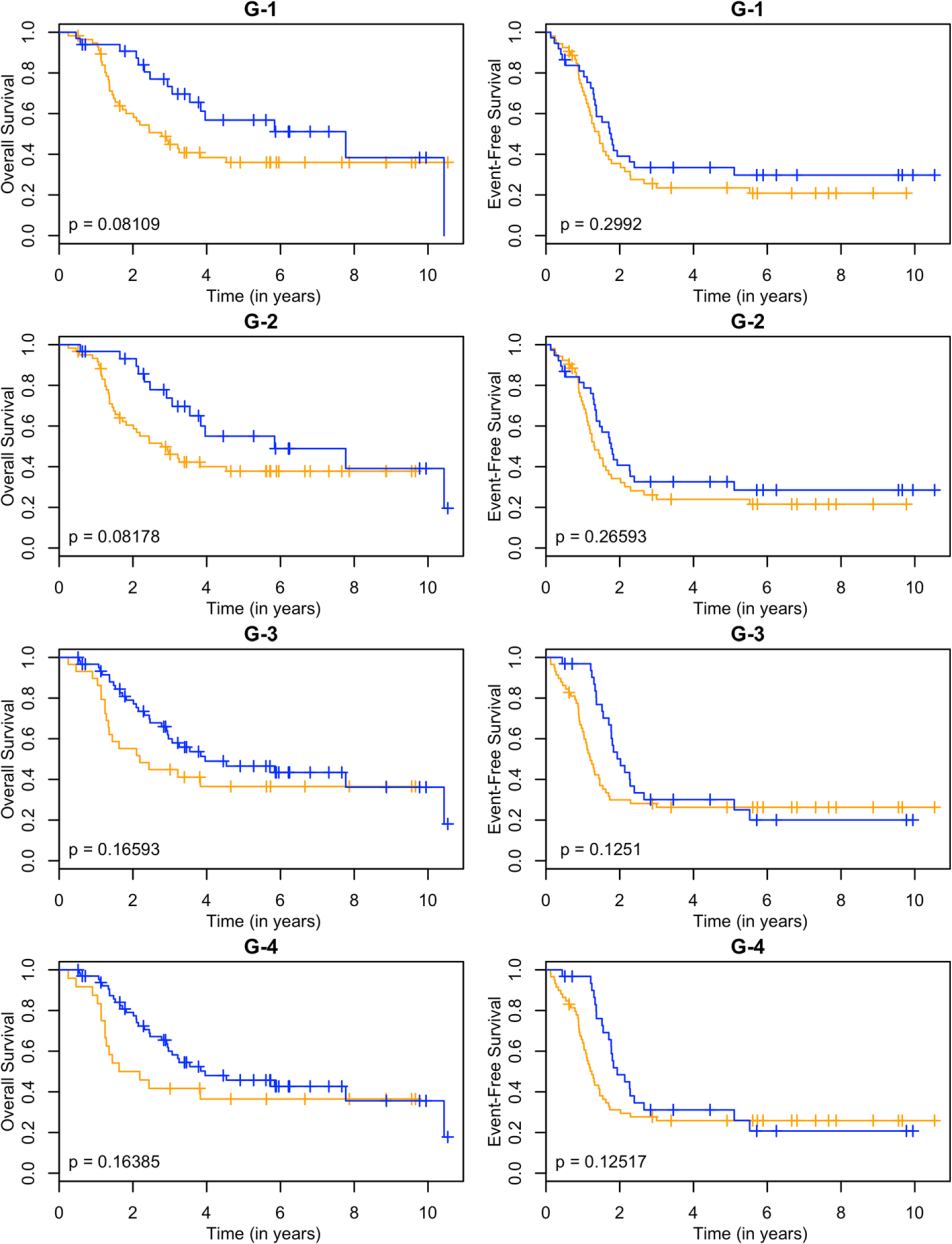
Kaplan-Meier estimates for HR and LPS2. Kaplan-Meier estimates for overall survival (left column) and event-free survival (right column) of clinically high risk patients using the gene annotation from the RNA-seq data. Rows 1-4 correspond to PLS, SPLS, lasso, and elnet fitting procedures. The orange line corresponds to patients labeled as LPS2 (predicted survival time less than 2 years), and blue lines are non-LPS2. The *p*-values are for the logrank test

**Fig. 4:**
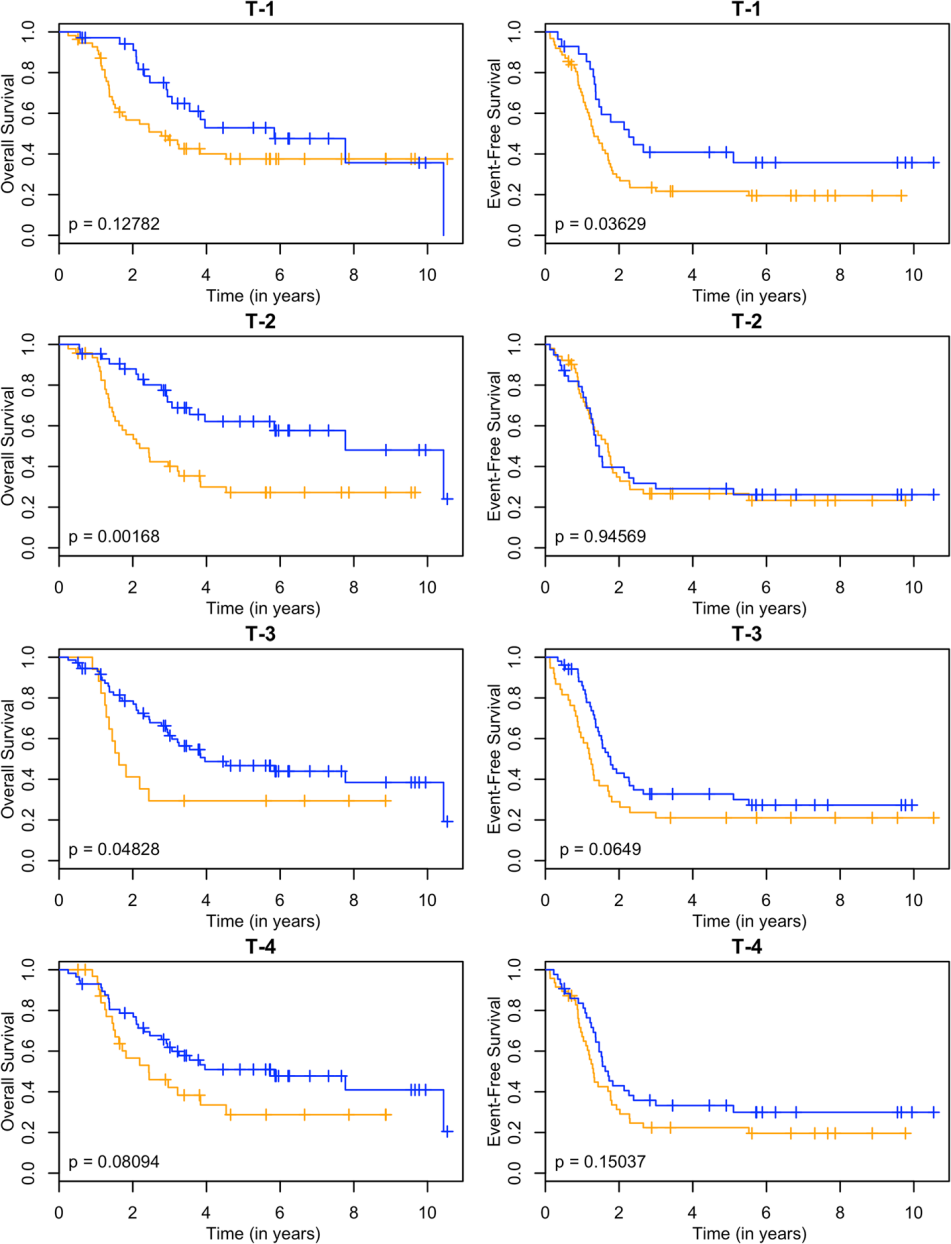
Kaplan-Meier estimates for HR and LPS2. Kaplan-Meier estimates for overall survival (left column) and event-free survival (right column) of clinically high risk patients using the transcripts annotation from the RNA-seq data. Rows 1-4 correspond to PLS, SPLS, lasso, and elnet fitting procedures. The orange line corresponds to patients labeled as LPS2 (predicted survival time less than 2 years), and blue lines are non-LPS2. The *p*-values are for the logrank test

**Fig. 5:**
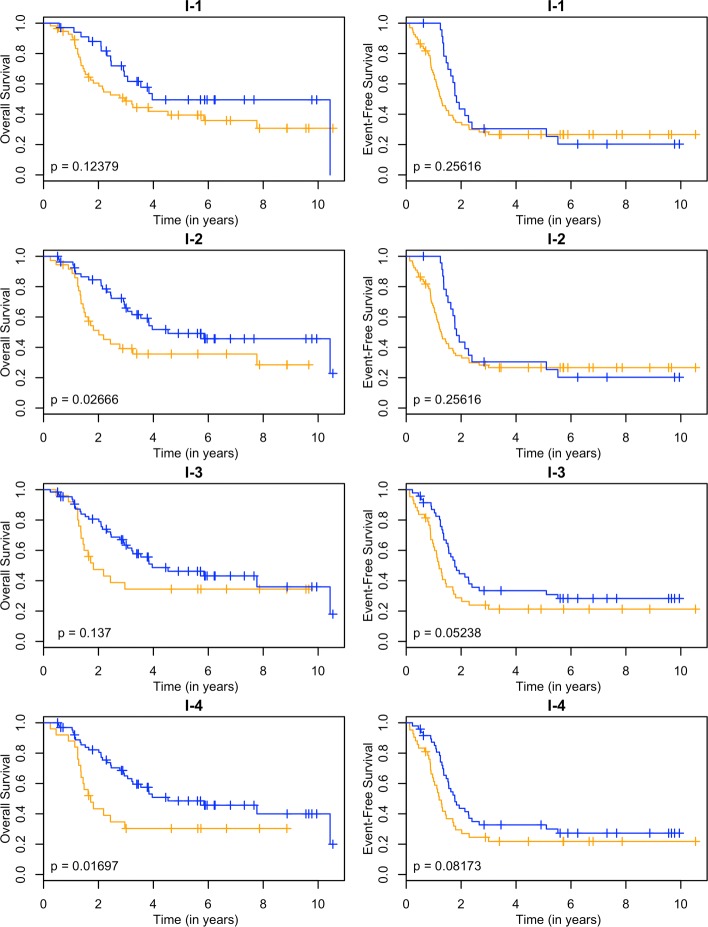
Kaplan-Meier estimates for HR and LPS2. Kaplan-Meier estimates for overall survival (left column) and event-free survival (right column) of clinically high risk patients using the introns annotation from the RNA-seq data. Rows 1-4 correspond to PLS, SPLS, lasso, and elnet fitting procedures. The orange line corresponds to patients labeled as LPS2 (predicted survival time less than 2 years), and blue lines are non-LPS2. The *p*-values are for the logrank test

**Fig. 6:**
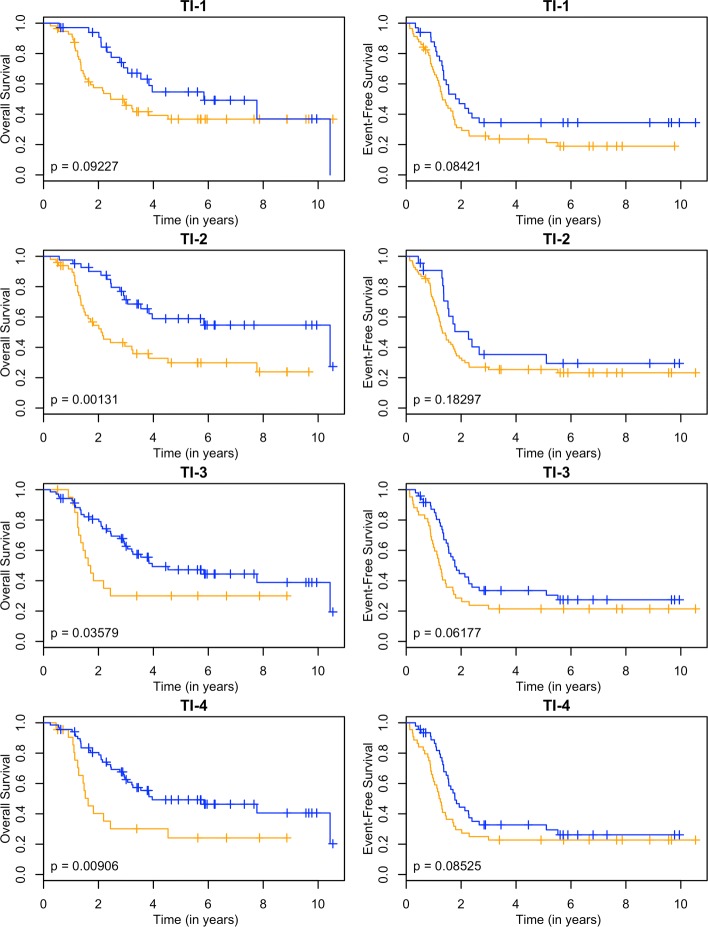
Kaplan-Meier estimates for HR and LPS2. Kaplan-Meier estimates for overall survival (left column) and event-free survival (right column) of clinically high risk patients using both the transcript and intron annotations from the RNA-seq data. Rows 1-4 correspond to PLS, SPLS, lasso, and elnet fitting procedures. The orange line corresponds to patients labeled as LPS2 (predicted survival time less than 2 years), and blue lines are non-LPS2. The *p*-values are for the logrank test

**Fig. 7:**
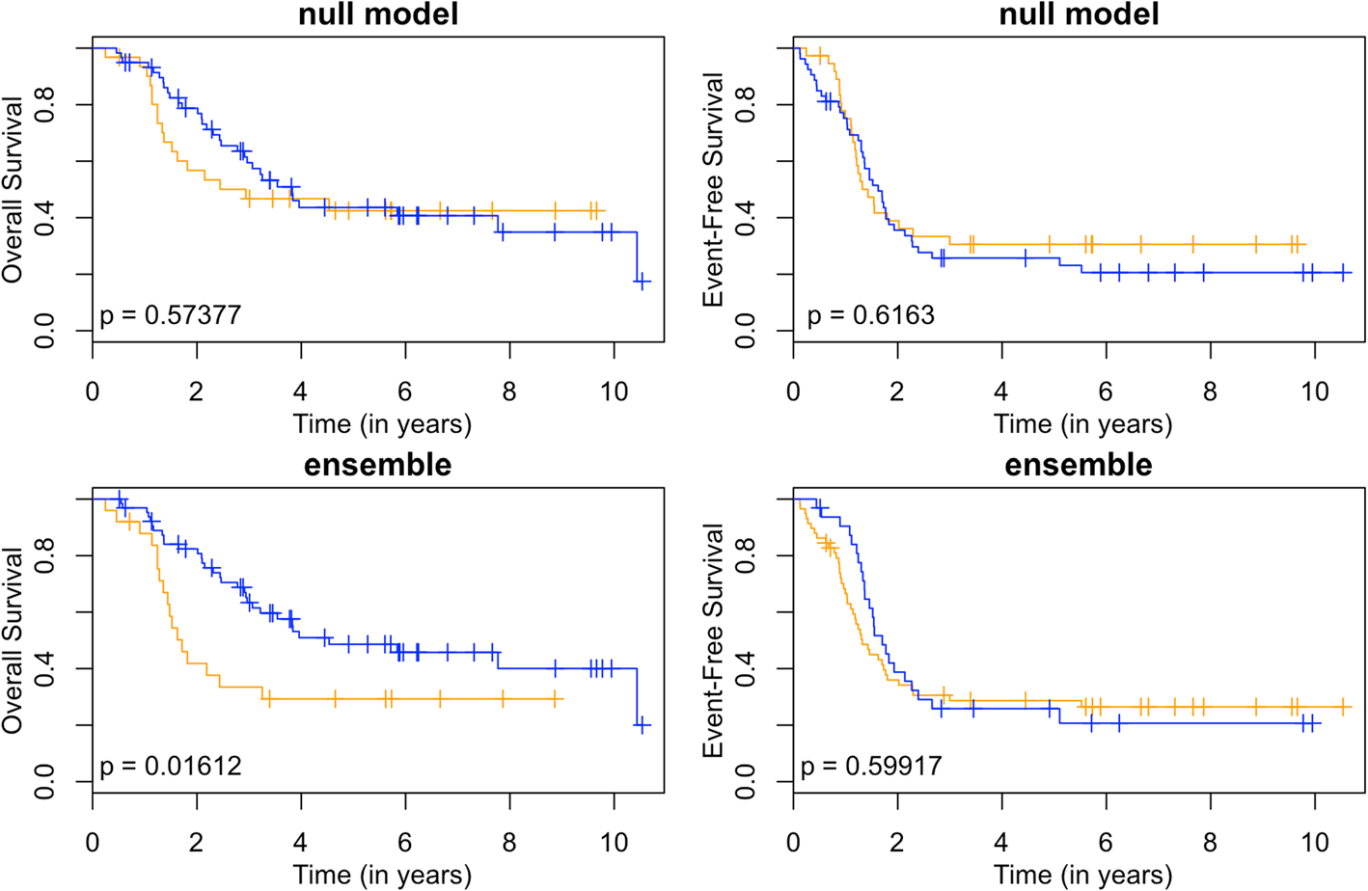
Kaplan-Meier estimates for HR and LPS2. Kaplan-Meier estimates for overall survival (left column) and event-free survival (right column) of clinically high risk patients using the null model (first row) and the ensemble approach (second row). The orange line corresponds to patients labeled as LPS2 (predicted survival time less than 2 years), and blue lines are non-LPS2. The *p*-values are for the logrank test

These models can be used as a classifier by comparing the predicted survival times to a chosen threshold. Since the clinically high-risk group is notorious for having poor prognosis, our goal is focused on subclassifying these patients. A threshold of 2 years is used. If a patient has a predicted survival time less than 2 years, they are labeled as LPS (low predicted survival). Otherwise, they are non-LPS. A classifier is considered successful if the two resulting groups (LPS versus non-LPS) have distinct survival curves. The Kaplan-Meier estimates [22] of these curves for each RNA-seq model are shown in Figs. 3, 4, 5 and 6, and the null model and ensemble are shown in Fig. 7.

Using OS as the outcome, almost every RNA-seq model is able to partition high-risk patients into two distinct groups, providing a substantial improvement over the null model. The TI-4 model produces groups with the largest difference in 2-year OS rates: 0.40±0.11 versus 0.80±0.05 (Table 1). With EFS as the outcome, there is less separation between LPS and non-LPS groups than is found with OS (Figs. 3, 4, 5 and 6). The T-1 model provides the largest distinction in 2-year EFS rates: 0.29±0.06 versus 0.56±0.10 (Table 1).

**Table 1:** Summary of Kaplan-Meier estimates for 2-year OS and 2-year EFS for clinically high-risk patients using each of the 18 proposed models

		LPS			non-LPS				
Outcome	Prob	SE	N	Prob	SE	N	Data	Model	*P*-value
OS	0.57	0.09	31	0.79	0.05	59	Null model		0.574
OS	0.60	0.07	57	0.91	0.05	33	G	1	0.081
OS	0.60	0.06	60	0.93	0.05	30	G	2	0.082
OS	0.55	0.09	29	0.79	0.05	61	G	3	0.166
OS	0.50	0.10	24	0.79	0.05	66	G	4	0.164
OS	0.57	0.07	55	0.94	0.04	35	T	1	0.128
OS	0.56	0.07	47	0.88	0.05	43	T	2	0.002
OS	0.41	0.12	17	0.78	0.05	73	T	3	0.048
OS	0.57	0.09	33	0.79	0.05	57	T	4	0.081
OS	0.61	0.07	56	0.88	0.06	34	I	1	0.124
OS	0.51	0.08	36	0.85	0.05	54	I	2	0.027
OS	0.47	0.10	25	0.81	0.05	65	I	3	0.137
OS	0.43	0.10	25	0.82	0.05	65	I	4	0.017
OS	0.57	0.07	56	0.94	0.04	34	TI	1	0.092
OS	0.54	0.07	49	0.90	0.05	41	TI	2	0.001
OS	0.40	0.11	21	0.81	0.05	69	TI	3	0.036
OS	0.40	0.11	22	0.80	0.05	68	TI	4	0.009
OS	0.42	0.10	25	0.82	0.05	65	Ensemble		0.016
EFS	0.39	0.08	37	0.36	0.07	53	Null model		0.616
EFS	0.35	0.07	53	0.39	0.08	37	G	1	0.299
EFS	0.34	0.07	52	0.41	0.08	38	G	2	0.266
EFS	0.30	0.06	58	0.50	0.09	32	G	3	0.125
EFS	0.31	0.06	59	0.48	0.09	31	G	4	0.125
EFS	0.29	0.06	62	0.56	0.10	28	T	1	0.036
EFS	0.35	0.07	51	0.40	0.08	39	T	2	0.946
EFS	0.29	0.07	38	0.43	0.07	52	T	3	0.065
EFS	0.31	0.07	47	0.43	0.08	43	T	4	0.150
EFS	0.35	0.06	66	0.43	0.10	24	I	1	0.256
EFS	0.35	0.06	66	0.43	0.10	24	I	2	0.256
EFS	0.29	0.07	43	0.45	0.07	47	I	3	0.052
EFS	0.29	0.07	42	0.44	0.07	48	I	4	0.082
EFS	0.31	0.06	57	0.47	0.09	33	TI	1	0.084
EFS	0.33	0.06	68	0.50	0.11	22	TI	2	0.183
EFS	0.29	0.07	42	0.45	0.07	48	TI	3	0.062
EFS	0.30	0.07	44	0.44	0.08	46	TI	4	0.085
EFS	0.36	0.06	58	0.39	0.09	32	Ensemble		0.599

Patients with predicted survival of less than 2 years are labeled as Low Predicted Survival (LPS), and otherwise are non-LPS. Columns labeled “Prob.”, “SE”, and “N” correspond to the estimated probability of 2-year survival, the standard error of the estimate, and the number of patients in the given cohort. The *P*-values are for the logrank test comparing LPS to non-LPS survival. The “Data” column refers to the type of RNA-seq data used, and the “Model” column refers to the dimension reduction technique used

In general, subclassification is more successful with OS than with EFS. The ensemble approach (Fig. 7) reflects the overall performance in both cases: the LPS and non-LPS groups are well separated by the ensemble in OS (0.42±0.10 versus 0.82±0.05) but not for EFS (0.36±0.06 versus 0.39±0.09) (Table 1).

### Pathway analysis

Pathway enrichment analysis provides a biological summary of the genes selected by the AFT model. Gene sets are constructed by collecting the predictors with nonzero coefficients in the fitted G-4, T-4 and TI-4 models. The I-4 model with introns only is not considered, since introns cannot easily be interpreted in the pathway analysis. The PLS and SPLS methods gave each predictor some weight in the AFT model, while the predictors selected by lasso are a subset of those selected by elnet. Hence, only models fit using elnet are considered, as these contain an amount of sparsity that is appropriate for pathway analysis. Two gene sets are constructed, one associated with OS and the other with EFS. Pathway enrichment analysis (on KEGG pathways) is performed using DAVID 6.8 [23] and summarized in Tables 2 and 3.

**Table 2:** Pathway enrichment analysis of genes selected by the G-4, T-4, and TI-4 models when predicting OS (no pathways were significantly enriched for EFS)

Outcome	Pathway	Count	Size	*P*-value	BH
OS	Pathways in cancer	26	393	<0.001	0.010
OS	ErbB signaling pathway	11	87	<0.001	0.012

The columns include KEGG pathway name, the number of genes in the gene set that are in the pathway, the total number of genes annotated for the pathway, the *p*-value from a modified fisher’s exact test, and the Benjamini-Hochberg corrected *p*-value

**Table 3:** Pathway enrichment analysis of genes selected by the G-4, T-4, and TI-4 models

Outcome	Pathway	Count	Size	*P*-value	BH
OS	ErbB signaling pathway	11	60	0.029	0.999
OS	Salivary secretion	6	23	0.042	0.995
OS	Inflammatory mediator regulation of TRP channels	9	48	0.049	0.983
EFS	Terpenoid backbone biosynthesis	4	8	0.010	0.906
EFS	Metabolic pathways	29	304	0.016	0.847
EFS	Valine, leucine and isoleucine degradation	5	20	0.032	0.911
EFS	Biosynthesis of antibiotics	12	98	0.037	0.882
EFS	Fatty acid metabolism	5	21	0.037	0.820

In this analysis, the GeneCards genes are used at the background. The columns include survival outcome (OS or EFS), KEGG pathway name, the number of genes in the gene set that are in the pathway, the total number of genes annotated for the pathway, the *p*-value from a modified fisher’s exact test, and the Benjamini-Hochberg corrected *p*-value

When predicting OS, a total of 354 unique genes are given nonzero coefficients by one of the three models. Of these genes, 186 are annotated in KEGG pathways. DAVID uses a modified fisher exact test to compute *p*-values for enrichment, and the Benjamini-Hochberg correction is applied to account for multiple testing [24]. Two pathways are found to be significantly enriched: Pathways in Cancer and ErbB signaling pathway (Table 2). For EFS, 246 unique genes have nonzero coefficients, of which 135 are annoted in KEGG pathways. However, no pathways are enriched for EFS at the 0.05 significance level.

The preceeding enrichment analysis uses the entire human genome as a background, which contains 6910 genes annoted in KEGG pathways. However, the RNA-seq data used in this study are filtered based on the GeneCards database. Hence, the pathway enrichment may be more appropriately conducted using those GeneCard genes as the background. The GeneCards database contained 3512 genes related to neuroblastoma, of which 2044 are annoted in KEGG pathways. Relative to this background, three pathways are enriched for OS: ErbB signaling pathway, Salivary secretion, and Inflammatory mediator regulation of TRP channels (Table 3). Five pathways are enriched for EFS: Terpenoid backbone biosynthesis; Metabolic pathways; Valine, leucine and isoleucine degradation; Biosynthesis of antibiotics; and Fatty acid metabolism (Table 3). These pathways have *p*-values below the 0.05 significance level, but are nonsignificant after applying the Benjamini-Hochberg correction.

## Discussion

In this study we used the AFT model, fit using various dimension reduction techniques and a dataset imputation procedure, to predict overall survival (OS) and event-free survival (EFS) times of neuroblastoma patients. Three feature levels of an RNA-seq dataset were considered, including genes, transcripts, and introns. Models were fit using the three features independently and with transcripts and introns together.

In terms of RMSE, the predictive performance of OS is greatly improved in the RNA-seq models over the null model, but this improvement is curtailed when predicting EFS. The high rate of censoring that is found in this data will be a hinderance for any nonparametric model. Alternative approaches can be considered: One possibility is to switch to semiparametric estimation, but this approach will be computationally intensive in this high-dimensional setting. A more practical solution may be to employ a boosting algorithm (see [25] for example). These alternatives were not explored in detail in this paper.

The second goal is to subclassify clinically high-risk (HR) patients. In this venture the AFT model produces very promising results. High-risk patients with low survival times are more sensitive to the amount of error remaining in predicted times, but the estimates tend to be in the right direction. That is, the relative ordering of the patients by their predicted survival times is accurate. A reviewer suggested the use of Harrell’s c-index [21] to measure this effect. The c-index is above 0.8 for each model when predicting OS, indicating strong concordance between predicted OS time and true OS times (Fig. 1). The concordance is less strong when predicting EFS (Fig. 2).

Using a cutoff of 2 years, each model is converted to a classifier. The TI-4 model provides the best results for OS. For EFS, the I-4 model appears to be the best. A classifier using 5 years as a cutoff is also considered, but the performance is not as good; setting the threshold to a value below 5 years seems to be necessary in order to identify those patients who are at the highest risk in the HR group.

A pathway analysis of the gene sets selected by the elastic net when predicting OS and EFS is performed. With OS, two cancer-related pathways are enriched. This analysis may be biased, however, since the RNA-seq data are initially filtered using the GeneCards database. If the background is altered to reflect this filtering, we find that one of the two cancer-related pathways remains relatively enriched. This alteration also reveals additional enriched pathways for the OS and EFS gene sets, but their relevance to neuroblastoma is questionable. Since the prediction of EFS had limited success, it is no surprise that the genes selected for EFS appear to have limited biological relevance.

The predictive accuracy and pathway enrichment for OS suggests that the AFT model with elastic net is able to pick out biologically meaningful genes. A future study pursuing this kind of interpretation will need to consider the stochastic nature of the fitting procedure and determine a stable set of genes selected by the model. As suggested by a reviewer, we can also explore relationships between these genes and those excluded by the initial filtering process. Such an investigation may produce biological insights into the subgroups of high-risk patients.

An ensemble of models was considered, which incorporates bagging with rank aggregation of three performance measures. The performance of the ensemble method is comparable to that of the best individual model. This suggests that the ensemble method is able to effectively combine models fit on separate datasets. If additional datasets are incorporated, such as copy number variation or other -omics data, the AFT model can be fit by simply concatenating the datasets together, but the computational requirement quickly becomes too burdensome. The ensemble approach may provide a useful heuristic for combining several datasets. We have shown that this heuristic works well in combining different annotations of RNA-seq data, but further investigation is needed to verify the performance with disparate datasets.

## Conclusion

In this study, we explored the performance of the AFT model in predicting survival times for neuroblastoma patients. A classifier was constructed by comparing predicted survival times to a 2-year threshold. Using both transcript and intron annotations in the model gave the best performance. We are able to subclassify clinically high-risk patients into two distinct groups, one with a 40% 2-year overall survival rate and the other at 80%. This suggests that the AFT model is useful in subclassifying high-risk patients, which can help clinicians in choosing effective treatment plans. Only RNA-seq data was considered in this study, but other types of data can be used as well. The ensemble method is a useful heuristic for combining several high-dimensional datasets under this framework, and it has been shown capable of maintaining optimal performance.

## Reviewers’ comments

### Reviewer’s report 1: Subharup Guha, University of Florida, Gainesville, USA

The authors explore the performance of the AFT model in predicting survival times for neuroblastoma patients. This is a very well-written paper. Overall, the analysis is scientifically compelling and relies on creative applications of sound statistical techniques. The classifier comparing the predicted survival times to a 2-year threshold is successful when it is based on transcript and intron annotations. The ensemble method and its potential application to fitting disparate datasets holds much promise for future work.

**Reviewer comment:** As a suggestion for future research, but entirely unrelated to the current paper which is more than satisfactory, I have the following suggestion. From the second paragraph of the Discussion, it appears that it may be helpful to explore Harrell’s C-index as an alternative measure of accuracy. This may be a better measure than RMSE for the parametric models, especially because they appear to get the relative ordering of the survival times right rather than the actual magnitudes.

Author’s response: *We thank Dr. Guha for this suggestion. The performance of each model using Harrell’s c-index has been added to the revised manuscript*.

**Reviewer comment:** On Line 7 of page 2, should the comma following INSS be deleted? 2. On Line 7 of page 6, what is K?

Author’s response: *Grammatical corrections have been made to the manuscript. For the latter point, there are K = 3 performance measures in this study. This is now clarified in the text*.

### Reviewer’s report 2: Isabel Nepomuceno, Universidad de Sevilla, Seville, Spain

In this paper, authors used the accelerated failure time (AFT) model with four dimension reduction techniques and a dataset imputation scheme to predict overall survival and event-free survival times of neuroblastoma patients. Three feature levels of and RNA-Seq dataset were considered. Authors shown that the use of RNA-Seq data improves accuracy in comparison to using clinical data alone. In general the paper is appropriate to the journal. The analysis presented in this paper is very interesting. I have several suggestions and comments to be revised:

**Reviewer comment:** The Method section is written in a clear manner but is difficult to reproduce. Authors mentioned the R package used but they don’t provide the R code of the study.

Author’s response: *We thank Dr. Nepomuceno for her comments and suggestions. All R code and output is available from GitHub at **https://github.com/tgrimes/CAMDA-2017-Neuroblastoma.** The session info is also reported, which includes the R version, computer specifications, and a list of the packages used during the analysis*.

**Reviewer comment:** The Ensemble Method subsection, authors use bagging with rank aggregation over each performance measure and set B to 20. Why this parameter is fixed to 20 should be explained. And authors should explain why the use bagging instead of cross validation.

Author’s response: *The choice of 20 iterations for bagging is a compromise between computation time and model performance. We also considered B = 50 but did not find a substantial change in performance*.

**Reviewer comment:** The description of the RNA-Seq Data, authors reduce the "raw data" with 60776 genes into 3401 using the 3681 genes related to neuroblastoma obtained from the Gene Cards Suite. Have authors made some analysis from the remaining genes? Could be genes related with the problem and not related with the disease? It could be interesting to do a cluster analysis to see if the grouped genes using prior knowledge are also clustered together in this analysis.

Author’s response: *These are interesting suggestions that deserve a separate analysis to be fully addressed. The main purpose in using the Gene Cards database was to provide an initial filtering to speed up computation. We also re-ran the analysis without this step and found little difference in predictive performance. We are careful not to place too much emphasis on the interpretation of the gene sets obtained in this analysis. As you’ve pointed out, there are many new questions that have been uncovered and deserve careful consideration. We’ve added some comments regarding this in the discussion section of the manuscript*.

**Reviewer comment:** Furthermore, a reference about the Cox proportional hazards model or the R package used should be added.

Author’s response: *We thank the author for pointing out this omission. The revised manuscript now contains additional references*.

**Reviewer comment:** Section Results, classification of high-risk patients should be rewritten. The second and third paragraph is confused and difficult to see which plot corresponds with each sentence.

Author’s response: *This section has been reworded to clarify which table or figure each sentence is referring to. The titles for each plot have been changed in concordance to the labels used to identify each model within the manuscript*.

**Reviewer comment:** In section Pathway analysis, authors claim that several genes are involved in several pathways. That means, do genes appear in the pathways or are the pathways enriched by the set of genes? If it is the second case, authors should add a table with the list of pathways, the number of entities in the pathways and the number of genes from the set which appear in the pathway.

Author’s response: *We thank the reviewer for prompting this clarification. Previously, the interpretation was that genes appear in the pathways. But this initial approach seems uninformative, particularly since we use the GeneCards database to subset on genes, which would bias our selection to genes in cancer-related pathways. In response, we have modified this section and now conduct a pathway enrichment analysis. However, a question is raised regarding the choice of background: should our gene sets be compared to all genes in the genome (as is usually done) or to the GeneCards genes that we subset on? With the former, there is a concern that the analysis may be biased. Results for both of these scenarios have been added to the manuscript*.

**Reviewer comment:** Finally, as minor comments: - The Bibliography Section must be revised, there are some incomplete reference as for example number 14. - In Table 1, one of the models is named simple for the baseline model. It should be names null model as authors explained before.

Author’s response: *The bibliography section has been corrected, and the tables and figures have been relabeled to be consistent with the text*.

## Abbreviations

AFT: Accelerated failure time
CI: Confidence interval
EFS: Event-free survival
elnet: Elastic net
HR: High-risk
INSS: International neuroblastoma staging system
lasso: Least absolute shrinkage and selection operator
LPS: Low predicted survival
OS: Overall survival
PLS: Partial least squares
RMSE: Root mean squared error
SPLS: Sparse partial least squares
